# Daily Intermittent Normobaric Hypoxia Over 2 Weeks Reduces BDNF Plasma Levels in Young Adults – A Randomized Controlled Feasibility Study

**DOI:** 10.3389/fphys.2018.01337

**Published:** 2018-10-01

**Authors:** Andreas Becke, Patrick Müller, Milos Dordevic, Volkmar Lessmann, Tanja Brigadski, Notger G. Müller

**Affiliations:** ^1^Institute of Cognitive Neurology and Dementia Research, Otto-von-Guericke-Universität Magdeburg, Magdeburg, Germany; ^2^Neuroprotection Laboratory, German Center for Neurodegenerative Diseases (DZNE), Magdeburg, Germany; ^3^Institute of Physiology, Otto-von-Guericke-Universität Magdeburg, Magdeburg, Germany; ^4^Center for Behavioral Brain Sciences, Magdeburg, Germany; ^5^Informatics and Microsystem Technology, University of Applied Sciences, Kaiserslautern, Kaiserslautern, Germany

**Keywords:** hypoxia, BDNF, neuroplasticity, IHT, adaptation

## Abstract

**Background:** The results from animal and human research indicate that acute intermittent hypoxia can enhance brain-derived neurotrophic factor (BDNF) plasma levels and gene expression. As BDNF is known to promote the differentiation of new neurons and the formation of synapses, it has been proposed to mediate adult neuroplasticity. Thus, the present study aimed to analyze the long-term effects of daily intermittent exposure to normobaric hypoxia (simulating high altitude exposure at approximately 4000–5000 m) over 2 weeks on BDNF levels in young adults.

**Methods:** Twenty-eight young adults (age: 19–33 years) were randomized into a hypoxic intervention group (*N* = 14) or the control group (*N* = 14). Participants in the intervention group breathed intermittent normobaric hypoxic air at resting conditions (5 min intervals, 80–85% SpO_2_ measured via a finger pulse oximeter, 12 sessions for 60 min/day for 2 weeks) via a hypoxic generator. BDNF plasma and serum levels were determined at baseline and at 2 weeks after intervention using sandwich ELISAs.

**Results:** After 2 weeks of daily intermittent hypoxic treatment (IHT), we found a significant group x time interaction effect for BDNF plasma levels based on a significant decrease in BDNF levels in the hypoxia group.

**Conclusion:** Our results demonstrate that daily intermittent administration of hypoxic air has a significant effect on BDNF regulation in healthy young adults. Contrary to other results reporting an increase in BDNF levels under hypoxic conditions, the present data suggest that hypoxic treatment using intensive IHT can reduce BDNF plasma levels for at least 2 weeks. This finding indicates that the daily application of hypoxic air is too frequent for the aimed physiological response, namely, an increase in BDNF levels.

## Introduction

Hypoxia is defined by a reduced oxygen content in air and can be divided into intermittent and chronic forms. Thereby, intermittent hypoxia applies to a large spectrum of stimuli that range from exercise in high altitude to obstructive sleep apnea (OSA). Intermittent hypoxia treatment (IHT) was first used in sports medicine to enhance human physical performance (erythropoiesis and angiogenesis) (Viscor et al., 2018). During the following years, hypoxic training was increasingly employed for non-pharmacological treatment of several diseases (e.g., bronchial asthma, hypertension, and cardiovascular diseases). IHT can effectively stimulate various metabolic processes (Serebrovskaya et al., 2008) and can have numerous positive health effects similar to cardiovascular physical activity (Enette et al., 2017). IHT may serve as a protective mechanism for the brain by inducing neurogenesis. For instance, histological studies in adult rats have shown that IHT promotes a transient increase in progenitor cell proliferation in the subventricular zone and a long-term increase in the dentate gyrus (Zhu et al., 2005) and has the potential to recover spatial learning deficits after cerebral ischemia by increased hippocampal neurogenesis (Tsai et al., 2011). However, intermittent normobaric hypoxia is not associated with positive effects only *per se*. For example, the clinical syndrome of OSA leads to intermittent hypoxia as well (Burtscher et al., 2009) and is associated with numerous negative effects such as reduced cognitive performance (Yan, 2014; Malle et al., 2016). Hence, based on different characteristics such as the dose and the duration, we can assume that hypoxia induces both protective and pathological effects. It has been proposed that low-dose intermittent hypoxia (9–16% inspired O_2_) with short durations can enhance positive physiological processes, whereby high-dose hypoxia (2–8% inspired O_2_) is associated with progressively pathological mechanisms (Navarrete-Opazo and Mitchell, 2014).

The results from animal and human research indicate that acute intermittent hypoxia (Vermehren-Schmaedick et al., 2012) and physical activity (Enette et al., 2017) can enhance brain-derived neurotrophic factor (BDNF) blood levels and BDNF gene expression. Such gene expression is explained by an oxygen deficit recognized by the oxygen sensory system (Sharp and Bernaudin, 2004) changing the oxygen-dependent degradation domain of hypoxia-inducible factor (HIF-1), thereby inducing an increase in HIF-1-alpha levels (Wiener et al., 1996). HIF-1-alpha is known to act as a transcription factor to modulate the expression of several genes, such as BDNF growth factor levels (Helan et al., 2014). The BDNF neurotrophin is a member of the nerve growth factor family and is widely expressed in the human brain, especially in the hippocampus, but it is also expressed in peripheral tissues such as the pulmonary vasculature (Aravamudan et al., 2012; Helan et al., 2014). Current research studies indicate BNDF plasma levels as a potential biomarker for reliable diagnosis of neurocognitive disorders (Levada et al., 2016). The protein is secreted in an activity-dependent manner but is also secreted in response to hypoxia (Haubensak et al., 1998; Hartmann et al., 2001; Kohara et al., 2001; Brigadski et al., 2005; Matsuda et al., 2009; Brigadski and Leßmann, 2014; Helan et al., 2014; Edelmann et al., 2015; Hartman et al., 2015). Research results indicate that 75% of the BDNF in the peripheral blood plasma originates from the brain (Krabbe et al., 2007; Rasmussen et al., 2009). Several studies have suggested that BDNF is an important modulator of the CNS and promotes the differentiation of new neurons and synapses (Huang and Reichardt, 2001; Leschik et al., 2013; Park and Poo, 2013; Edelmann et al., 2014). BDNF, therefore, represents one of the major mediators of neuroplasticity (Calabrese et al., 2014). Furthermore, some authors have suggested that BDNF blood levels may serve as a biomarker for the diagnosis of neurodegenerative diseases and psychiatric disorders and can also serve as a surrogate marker for the success of therapies in these disorders (Ruscheweyh et al., 2011). Reduced BDNF blood levels have been reported in Alzheimer’s disease (Laske et al., 2007) and mild cognitive impairment (Forlenza et al., 2010).

Regarding the effect of intermittent hypoxia on BDNF blood levels in humans, the status of research is currently unclear. The results from animal and human studies have shown an acute increase in BDNF plasma levels in response to hypoxia. Helan et al. (2014) observed an increase in BDNF levels in 30 healthy volunteers after 72 h of normobaric hypoxia. Schega et al. (2016) reported no effects on BDNF in serum in older adults (*N* = 34, 66.4 ± 3.3 years) after 4 weeks of intermittent normobaric hypoxia (3× per week for 90 min) in addition to cardiovascular exercise. However, their data indicated that BDNF levels increased in the exercise-intervention group and in the exercise control-group after a compensation period of several weeks. This finding raises the question of whether the delayed effect could have been observed after hypoxic treatment alone, i.e., without concomitant cardiovascular exercise intervention.

Previous studies in animal research indicate an occurrence of neurogenesis in dentate gyrus within 4 weeks subsequent to intermittent hypoxia (Zhu et al., 2005). Based on these results we conducted a feasibility study to test the effects of 2 weeks of daily exposure to hypoxic air, which simulated intermittent hypoxia treatment (IHT), on peripheral BDNF levels. Therefore, we expected an increase in BDNF levels (as a central mediator of neurogenesis).

With respect to previous research on passive IHT methods, a protocol was chosen that has been shown to increase aerobic capacity and exercise tolerance in elderly men (Burtscher et al., 2004). In view of the data from Zhu et al. (2005) and based on recommendations for IHT regimes (Bassovitch and Serebrovskaya, 2009), we estimated the peak long-term effects of IHT to emerge 2 weeks after the intervention. If successful, this process is an easy to administer, low-cost intervention that may have great potential in inducing neuroplasticity and preventing cognitive deficits.

## Materials and Methods

The study was designed as a two-week randomized, controlled intervention. The ethics committee at the Otto-von-Guericke-Universität Magdeburg, approved the study, and all of the subjects signed a written informed consent form prior to participation. The exclusion criteria were acute or chronic cardiovascular, renal, metabolic, orthopedic and/or neurological diseases.

Twenty-eight young adults (age: 19–33 years) were randomized to a hypoxic intervention group [*N* = 14 (9 female), mean age 27.78, *SD* = 2.39] or a control group [*N* = 14 (5 female), mean age 22.85, *SD* 2.35] using the website www.randomization.com. The participants in the intervention group breathed intermittent normobaric hypoxic air at resting conditions (5 min intervals at a target of 80–85% SpO_2_ via a finger pulse oximeter, 12 sessions for 60 min/day for 2 weeks) generated by a hypoxic generator (b-cat and integra ten). The simulated high altitude was continuously manually adjusted between 4000 and 5000 m to reach the target SpO_2_. The control group received no intervention.

Fasting blood samples were taken in the mornings at baseline and at posttest (2 weeks after the last training session). From the blood samples, the plasma and serum concentrations of BDNF were determined using sandwich ELISAs (BDNF DuoSet; R&D Systems, Wiesbaden, Germany) as previously described (Schega et al., 2016).

For the intervention group, the blood samples for the small blood count were taken 4 times at baseline, 1 week after the intervention, at the end of intervention (consecutive day of last intervention session) and 2 weeks after the intervention. Five missing data sets for the second time point and 3 missing data sets for the third time point were reported (subjects did not show up).

Statistical analysis of BDNF plasma levels, BDNF serum levels and small blood count levels were performed with SPSS (SPSS 22 Inc./IBM). The intervention effects for BDNF were tested using repeated-measures ANOVAs with group (IHT and CG) as the between-subject factor and time (pre and post) as the within-subject factor. Age and gender were included as covariates. Additionally, *post hoc* pairwise comparisons were performed to determine the longitudinal changes in the hypoxia and control groups separately. In the case of non-normal distribution of data, we used the Mann-Whitney U-test or the Wilcoxon test instead of *t*-tests. The effect size was quantified by partial eta squared (*η*^2^). For interaction effects, the percentage changes from baseline to post measures were calculated for BDNF and small blood count values and were then correlated with Pearson’s formula.

## Results

**FIGURE 1:**
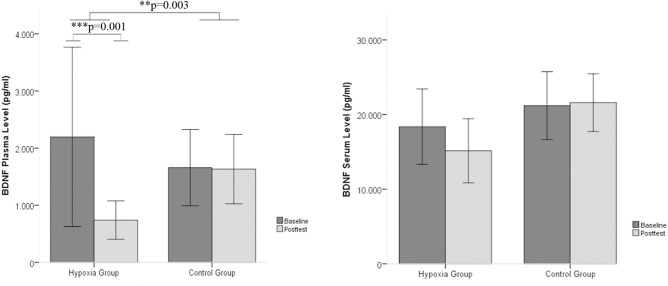
BDNF plasma and serum levels between baseline and post intervention measures in the hypoxia intervention group and the control group (Mean ± SD). ^∗∗^*p* < 0.01 and ^∗∗∗^*p* < 0.001.

The plasma and serum levels of BDNF were analyzed in the blood samples before the onset of the intervention as well as after the intervention. A significant group x time interaction effect was observed for the BDNF plasma levels [*F*(1,26) = 10.742, *p* = 0.002, *η*^2^ = 0.292]. *Post hoc* pairwise comparisons showed a significant decrease in BDNF plasma levels only in the hypoxia group from baseline to the posttest period (Wilcoxon-Test, *Z* = -3.296, *p* = 0.001). The intraindividual changes in BDNF plasma levels reached a reduction of 66.34% of the pretreatment period. No significant time × group interactions emerged for BDNF serum levels (see **Table 1** and **Figure 1**).

**Table 1:** Statistics of rANOVA on BDNF plasma and serum levels.

		Time			Group			Interaction (time x group)		
BDNF	*df*	*F*	*P*	*η*^2^	*F*	*p*	*η*^2^	*F*	*p*	*η*^2^
plasma level	1,26	11.52	0.002^∗∗^	0.307	0.425	0.520	0.016	10.742	0.003^∗∗^	0.292
Serum level	1,25	2.24	0.147	0.082	10.53	0.003^∗∗^	0.296	3.68	0.066	0.128

*^∗∗^*p* < 0.01*.

For the intervention group, blood samples for a small blood count were collected 4 times at baseline, 1 week after the intervention, at the end of the intervention (consecutive day of the last intervention session) and 2 weeks after the intervention. Five missing data sets for the second time point and 3 missing data sets for the third time point were reported (subjects did not show up). Using mixed linear effects to model the effect over time, the red blood cell distribution showed a linear decrease over time (*p* < 0.01; **Table 2**).

**Table 2:** Small blood count for the treatment group.

	Small blood count											
	Baseline (*N* = 14)		1 week IHT (*N* = 9)		2 weeks IHT (*N* = 11)		Posttest (*N* = 14)		Linear time effect		Quadratic time effect	
	Mean	SD	Mean	SD	Mean	SD	Mean	SD	*F*	*p*	*F*	*p*
RBCC (×10^6^/ml)	4.94	0.43	5.00	0.29	4.75	0.36	4.83	0.36	1.066	0.308	0.628	0.539
RDW (%)	13.66	0.90	13.64	0.13	12.70	0.62	12.61	0.59	20.84	0.00^∗∗^	1.259	0.295
HCT	0.46	0.03	0.44	0.03	0.43	0.03	0.45	0.03	1.852	0.181	1.443	0.248
HBG (mmol/L)	9.07	0.72	9.22	0.81	8.89	0.62	8.74	0.58	2.120	0.153	0.318	0.729
MCH (fmol)	1.84	0.13	1.84	0.08	1.87	0.10	1.81	0.09	0.287	0.595	0.903	0.414
MCHC (mmol/l)	19.79	0.79	21.02	0.55	20.48	0.52	19.70	0.53	0.163	0.689	11.77	0.000^∗∗^
MCV (fl)	93.14	3.80	87.40	2.79	91.64	4.37	92.07	4.08	0.145	0.705	3.866	0.029^∗^
WBC (×10^3^/ml)	8.22	3.72	7.72	1.80	7.28	0.75	6.21	0.80	5.099	0.030^∗^	0.106	0.899
SumGranul	0.63	0.10	0.66	0.06	0.61	0.06	0.58	0.06	3.369	0.074^t^	0.603	0.552
Segs	0.60	0.10	0.63	0.05	0.58	0.07	0.55	0.06	3.839	0.057^t^	0.908	0.411
Mono	0.06	0.01	0.07	0.01	0.06	0.02	0.06	0.02	0.071	0.792	0.496	0.613
Thrombocyte (×10^3^/ml)	285.14	78.16	278.80	41.56	271.91	66.43	252.64	42.76	1.883	0.178	0.074	0.929

*The mean values and SD for the RBCC, red blood cell count; RDW, red blood cell distribution width; HCT, hematocrit; HBG, hemoglobin; hypEryth, hypochromic erythrocytes; WBC, leukocyte count; MCH, lymphocytes, mean corpuscular/cellular hemoglobin; MCHC, mean corpuscular hemoglobin concentration; MCV, mean corpuscular volume; Mono, absolute monocyte count; Segs, segmented neutrophil granulocytes; SumGranul, sum granulocytes, thrombocytes. ^∗∗^*p* < 0.01 ^∗^*p* < 0.05 ^*t*^*p* < 0.09*.

Furthermore, an analysis of Pearson correlations between the baseline to post measure changes (%) revealed a close to significant positive correlation (one-tailed) for BDNF plasma and leucocyte counts (R_WBC_ = 0.446; *p* = 0.055) and a trend for a negative BDNF and lymphocyte interaction (R_lym_ = -0.374; *p* = 0.094).

## Discussion

Normobaric hypoxia such as with high-altitude training is generally assumed to have positive effects on physical and cognitive performance. Here, we tested the effect of a daily intermittent normobaric hypoxic training during a period of 2 weeks on the BDNF levels. While we observed the expected effects on blood parameters such as on the mean corpuscular hemoglobin concentration, contrary to our expectation, we found BDNF plasma levels to be significantly reduced 2 weeks after daily intermittent normobaric hypoxia over a period of 2 weeks. Regarding BDNF serum levels, no changes were detected. Research results from Pan et al. (1998) indicate that BDNF can pass the blood brain barrier by a high-capacity, saturable transport system and that 75% of BDNF plasma levels stems from the brain (Krabbe et al., 2007; Rasmussen et al., 2009).

Decreased BDNF levels are typically found in animal research when the animals have previously experienced stress. Various types of stress, including oxidative stress, have been shown to lead to decreased BDNF gene expression in cortical regions, including the hippocampus (Smith, 1996; Smith and Cizza, 1996; Bath et al., 2013; Kwon et al., 2013; Rothman and Mattson, 2013). In humans, a reduction in BDNF levels was seen after muscle damage or with very intensive physical exercise. To avoid such overtraining, successful exercise training is known to require sufficient resting periods (Parra et al., 2000). In rodents, physical activity induces BDNF gene expression in cortical regions, especially in the hippocampus (Neeper et al., 1995; Uysal et al., 2015). Studies on humans have reported an increase in BDNF levels following sportive interventions (Erickson et al., 2012; Müller et al., 2017a,b; Rehfeld et al., 2018). Others, however, failed to show changes in the levels of any of the neurotrophic factors that were assessed (Maass et al., 2016). A current review by Enette et al. (2017) provides a comprehensive analysis of the effects of aerobic training on BDNF plasma and serum levels in older adults. In 11 of the 14 randomized controlled trials that were included, the authors reported significantly increased BDNF plasma and/or serum levels after aerobic intervention.

Together, these findings indicate that our IHT protocol with its daily applications of hypoxic air might have been too intensive and, therefore, too stressful for the participants’ bodies. In agreement with this finding, we observed a change in blood marker levels that were indicative of inflammation, namely, lymphocytes and granulocytes. Intensive physical exercise also induces inflammatory processes (Brown et al., 2015), and the latter has also been shown to relate to reduced BDNF levels after acute exercise at higher intensities (Nofuji et al., 2012; Cabral-Santos et al., 2016). Other conditions in which a reduction in BDNF levels was observed in the past include sleep apnea (Wang et al., 2012), birth stress associated with psychiatric disease later in life (Cannon et al., 2008), and stroke with low functional outcome (Lasek-Bal et al., 2015). With respect to the present study, the results of Wang et al. (2017) are of special relevance, as sleep apnea is associated with nocturnal intermittent hypoxia. Again, this finding suggests that “overdosing” hypoxia has detrimental effects on BDNF secretion.

The assumption that our IHT protocol was too intense and therefore decreased BDNF levels leads to the crucial question of whether other less stressful IHT protocols could still have a positive effect. In addition, methodological aspects (sampling time and preanalytical variations) could have an influence on the gained results. Indeed, there is an ongoing discussion of what type of hypoxia treatment is most effective (Serebrovskaya and Xi, 2016). A protocol that increases physical fitness at the same time may have negative effects on BDNF (Enette et al., 2017). Indeed, we had used a protocol that, in a former study, had shown positive effects on aerobic capacity.

### Metabolic and Cardiovascular Response to Hypoxia

Several field experiments in the mountains and environmental studies in chambers report physiological effects of hypoxia (Heinonen et al., 2016). These experiments show that hypoxia can induce cardiovascular stress, can increase sympathetic neural activation and can alter energy metabolism. The complex metabolic response causes a release of various stress hormones (Kayser and Verges, 2013). Regarding cardiovascular response to normobaric hypoxia Heinonen et al. (2014) reported a significantly increased cardiac output, ejection fraction and tachycardia. Additionally, Heinonen et al. (2014, 2017) discuss hypoxia as a potential trigger for the release of brain natriuretic peptide (BNP) and the hormone apelin.

### Limitations and Outlook

This randomized controlled feasibility study has several limitations. First, the sample size was small (*N* = 28). Second, the blood samples were only analyzed at baseline and after 2 weeks of intervention. Another limiting factor in the BDNF blood analyses is the large variances.

Future studies are needed to evaluate the correct dose of normobaric intermittent hypoxia to increase BDNF plasma levels and examine the underlying neurobiological mechanisms. An intensive assessment (neuropsychology, MRI/PET, cortisol, and IGF-1) would be useful to analyze the physiological adaptations to hypoxia.

In addition, BDNF has been suggested to play a mediating role in schizophrenia (Sokoloff et al., 2004; Guillin et al., 2007). Thus, several studies indicate an increase of the BDNF levels and gene expression in patients with schizophrenia (Laske and Eschweiler, 2006). In conclusion, an intermittent normobaric hypoxia regimen that successfully increases the BDNF levels may offer a non-pharmacological treatment to patients with schizophrenia.

## Ethics Statement

This study was carried out in accordance with the recommendations of Ethics Committee of the Medical Faculty at the Otto-von-Guericke-Universität Magdeburg with written informed consent from all subjects. All subjects gave written informed consent in accordance with the Declaration of Helsinki. The protocol was approved by the Ethics Committee of the Medical Faculty at the Otto-von-Guericke-Universität Magdeburg.

## Author Contributions

AB contributed to study organization, data analysis, paper writing, and paper reviewing. PM contributed to data analysis, paper writing, and paper reviewing. MD reviewed the paper. VL and TB contributed to data analysis and paper reviewing. NM contributed to study organization, paper writing, and paper reviewing.

## Conflict of Interest Statement

The authors declare that the research was conducted in the absence of any commercial or financial relationships that could be construed as a potential conflict of interest.
